# Emergent Treatment of Neuroleptic Malignant Syndrome Induced by Antipsychotic Monotherapy Using Dantrolene

**DOI:** 10.5811/cpcem.2018.11.39667

**Published:** 2019-01-04

**Authors:** Vivian Ngo, Alfredo Guerrero, David Lanum, Michelle Burgett-Moreno, Gregory Fenati, Steven Barr, Michael M. Neeki

**Affiliations:** * Arrowhead Regional Medical Center, Department of Family Medicine, Colton, California; † Arrowhead Regional Medical Center, Department of Emergency Medicine, Colton, California; ‡ California University of Sciences and Medicine, Department of Emergency Medicine, Colton, California; § Arrowhead Regional Medical Center, Department of Anesthesia, Colton, California

## Abstract

Neuroleptic malignant syndrome (NMS) is a rare but potentially fatal complication resulting from neuroleptic drug therapy. Presentation of NMS can vary, and diagnosis relies primarily upon medical history and symptomatology. Due to the potential delay in diagnosis, emergency physicians should remain vigilant in recognizing the symptoms of NMS and be prepared to initiate immediate treatment following diagnosis. Dantrolene, which has been used for spasticity and malignant hyperthermia, has been reported as a potential treatment for NMS and led to off-label use for NMS. We report two cases of NMS induced by antipsychotic monotherapy for which dantrolene was administered.

## INTRODUCTION

Neuroleptic malignant syndrome (NMS) is a rare but potentially fatal complication of antipsychotic drug therapy.^1^,^2^ It occurs in 0.002–3% of patients taking neuroleptic drugs, including patients of both genders and all age groups.^3^,^4^ It is characterized by hyperthermia, blood pressure fluctuation, muscle rigidity, altered mental status, and tachycardia.^3^ Lab values typically show leukocytosis, elevated transaminases, metabolic acidosis, myoglobinuria, elevated creatine and blood urea nitrogen levels. Electrocardiogram (ECG) changes can reveal prolonged PR, QRS, and QT intervals as well as ST and T-wave abnormalities.^3^ The presentation of NMS is varied and may include some or all of these criteria.

Although formal diagnostic criteria have been proposed (Table 1), there is no universally-accepted criteria; the diagnosis of NMS is one that relies heavily on history and symptomatology rather than laboratory and diagnostic testing.^4^–^6^ Once the diagnosis of NMS has been suspected, treatment should be immediately initiated following current available guidelines to reduce morbidity and mortality associated with delays in treatment.^7^ However, while removal of the causative agent and supportive care are widely accepted steps in the management of NMS, the efficacy of specific medical treatments remains controversial.^8^,^9^ Most of the current guidelines are supported by clinical experience and case reports of patients treated for NMS due to the rarity of NMS presentation.^8^,^10^

There are a growing number of case reports on NMS, likely in relation to the ongoing use of antipsychotic monotherapy (Table 2).^1^,^7^,^11^–^17^ We present two cases of NMS induced by antipsychotic monotherapy treated with dantrolene in the emergency department (ED). Both cases were approved by the Arrowhead Regional Medical Center Institutional Review Board. The goal of this case report is to increase awareness of NMS among emergency physicians and to add to the growing body of knowledge surrounding NMS presentation and treatment options.

## CASE REPORT

### Case One

A 48-year-old male with past medical history of hypertension, diabetes, and schizophrenia was brought to the ED for acute altered mental status and combative behavior at home. Family reported a history of hallucinations and a recent medication change to haloperidol (Table 3). In the ED the patient presented lethargic with a Glasgow Coma Scale (GCS) of 4, foaming from the oropharynx, and rigid. Vitals included an intravesical temperature of 109.6°F, blood pressure of 143/129 millimeters mercury (mmHg), pulse of 133 beats per minute (bpm), respiratory rate of 12 breaths per minute and irregular, and 100% saturation on high oxygen flow via nasal cannula. The patient’s total creatine kinase was 28.482 units per liter (U/L) and troponin of 0.75 nanogram per milliliter (ng/mL) with ECG revealing lateral depressions.

The patient was intubated for airway protection and immediately cooled with evaporative cooling measures. Additionally, the patient’s rhabdomyolysis was managed with vigorous hydration. The cardiology team determined the patient was not stable enough for urgent cardiac catheterization, and heparin drip was started. Given the patient’s hyperthermia and muscle rigidity, NMS was suspected and an intravenous one milligram per kilogram (mg/kg) bolus dantrolene was administered in the ED. He was admitted to the intensive care unit (ICU) with a diagnosis of NMS, rhabdomyolysis, respiratory failure, and non-ST-elevation myocardial infarction. The ICU treatment team began bromocriptine at a dose of 2.5mg per nasal gastric tube every six hours per neurology recommendations and cooling through Arctic Sun 5000 Temperature Management System™.

**Figure 1:**
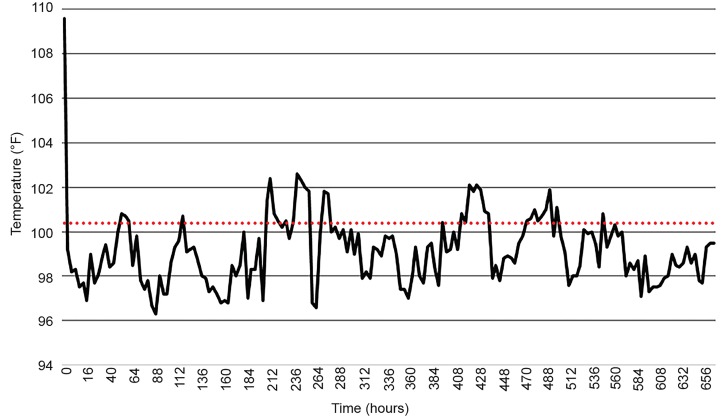
Patient one temperature vs. time.

He also experienced multi-organ insult including hepatic shock and acute renal failure. Furthermore, he continued to experience labile temperatures with episodic fevers (Figure 1). However, blood and urine cultures and cerebrospinal fluid (CSF) analysis were unremarkable. Additionally, he had acute loss of consciousness with wavering mentation, likely secondary to toxic metabolic encephalopathy, with GCS ranging from 4–11. After several failed multiple attempts for extubation, the patient subsequently required tracheostomy. He was discharged to an extended care facility for tracheostomy care after four weeks of inpatient management.

### Case Two

A 21-year-old male with past medical history of autism and psychiatric disorder on risperidone was brought to the ED by his family with concern of altered mental status. The family reported that the patient had been somnolent, nonverbal, febrile, and had developed an unsteady gait. The patient was presenting one-week post treatment with a depot dose of risperidone (Table 4). In the ED, the patient was nonverbal with a GCS of 11, rigid, somnolent but able to follow basic commands, and displayed masked facies. Vitals included a temporal temperature of 99.3°F, blood pressure of 146/97 mmHg, pulse of 125 bpm, respiratory rate of 20 breaths per minute and oxygen saturation of 98% at ambient air. Laboratory examination was significant for creatine kinase 1092 U/L. Urine drug screen, comprehensive metabolic panel, hematology, and CSF analysis were unremarkable. The patient’s ECG revealed sinus tachycardia with a heart rate of 102 bpm but was otherwise normal.

**CPC-EM Capsule**

What do we already know about this clinical entity?

*Neuroleptic malignant syndrome (NMS) is a rare but potentially fatal disorder with a variable onset following neuroleptic treatment*.

What makes this presentation of disease reportable?

*Patients suffering from NMS often present to emergency departments for treatment. Unfortunately, NMS is widely variable both in presentation and patient outcomes*.

What is the major learning point?

*Timely treatment of acute onset of NMS is essential to counteract the potential deleterious side effects of NMS. Dantrolene may also present an additional tool to improve long-term outcomes*.

How might this improve emergency medicine practice?

*Emergency physicians can reduce the long-term health impacts following NMS by rapidly recognizing its symptoms and expediting treatment, which may include dantrolene in appropriate cases*.

**Figure 2:**
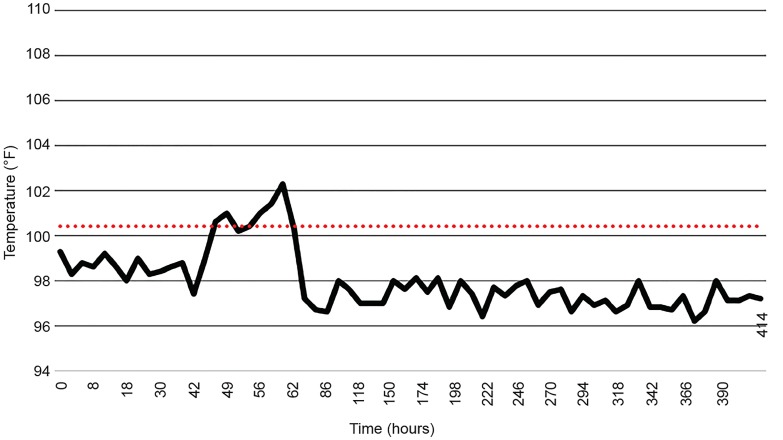
Patient two temperature vs. time.

The patient was started on an intravenous 1mg/kg bolus of dantrolene, followed by 1mg/kg intravenously every six hours in the ED. He was admitted to the ICU for close monitoring. On hospital day two, he began to show improvement in alertness and cognition but remained mostly somnolent with no improvement in muscle rigidity, and he spiked a fever of greater than 102.3°F (Figure 2). After four days, the intensive care team and neurology adjusted the dantrolene regimen to 40mg intravenously every six hours. Dantrolene was discontinued after 15 days and he was started on a 2.5mg dose of bromocriptine twice per day, which was subsequently adjusted to 5mg every eight hours.

By hospital day six, he was following basic commands and showing progressive symptomatic improvement. The patient continued to improve and by hospital day 10 demonstrated significant improvement in both gross and fine motor skills. He was subsequently discharged on hospital day 18 with continued bromocriptine treatment and close follow-up.

## DISCUSSION

Recent literature suggests that both diagnosis and treatment of NMS remain challenging.^11^,^18^ While NMS is primarily recognized by hyperthermia and peripheral muscle rigidity, presentation can often vary. Additional symptoms may include altered mental state, irregular blood pressure, tachycardia, metabolic acidosis, elevated creatine kinase, etc.^10^ In our case series, one patient presented with the most frequently reported NMS symptoms, hyperthermia and rigidity, while the second patient presented with only a mild fever and rigidity but also had an altered mental state, elevated blood pressure and elevated creatinine kinase, which led to the NMS diagnosis. Therapeutic approaches tend to vary as they are often implemented in a trial fashion rather than evidence-based practice.^10^

Primary treatment consists of withdrawal of the neuroleptic and supportive therapy such as hydration and cooling measures (Table 5).^8^ Medical therapy using dantrolene was first used for NMS in 1981 and has been cited in several reports as a useful treatment for NMS, particularly if the neuroleptic was a monotherapy.^10^ However, some studies have reported that dantrolene offers no significant benefit when compared to supportive therapy alone. Due to the low incidence rate of NMS, a randomized, controlled trial has not been feasible leading to a continued lack of consistency in treatment.^10^

There are several differences between our two cases that reveal firsthand the lack of consistency in treatment guidelines. The first patient received a bolus of dantrolene before being transferred immediately to the ICU for further management. Within the ICU setting, neurology and the intensive care team discontinued dantrolene and started bromocriptine. In contrast, the second patient remained in the ED for 12 hours and received two doses of dantrolene before being transferred to the ICU where dantrolene was continued until hospital day 15. He then was switched to bromocriptine. The differences in medical therapy seem to result from general practice differences between departments and specialists. Whereas dantrolene is commonly used for spasticity and malignant hyperthermia by ED providers, neurologists and intensivists favor bromocriptine as the medical therapy for management of NMS. This change in medical therapies used for NMS management during a hospital course has been reported previously.^1^,^11^

The common theory of pathogenesis of NMS is dopamine blockade with subsequent disruption of the hypothalamus and corpus striatum, leading to temperature deregulation and muscle contractions.^19^ Dopamine pathways play a crucial role in hypothalamus function and temperature regulation, which can be disrupted by dopamine receptor antagonists such as haloperidol and risperidone.^20^ Derangement of hypothalamic function can cause hyperthermia, arrhythmias, and irregular blood pressure and respiration. Additionally, dopamine blockade in the corpus striatum can lead to increased muscular rigidity and may lead to the occurrence of non-traumatic rhabdomyolysis. A recent study found that rhabdomyolysis and acute kidney injury were the most common complications in NMS – 30.1% and 16.1%, respectively.^21^ Additionally, acute kidney injury was associated with 2.3 times increased odds of death.^21^

Although the use of dantrolene in the setting of NMS is widely reported due to its effectiveness in spasticity and malignant hyperthermia, its pathophysiological effects are unclear. It is known that dantrolene directly relaxes skeletal muscle by inhibiting both ryanodine receptor binding and calcium release from intracellular storage in the sarcoplasmic reticulum. As an inhibitor of calcium release, it is suggested that dantrolene may also treat or prevent neuronal injury.^22^ The binding of dantrolene to ryanodine receptors in the brain protects neurons from the disruptions in calcium that occur in NMS. There is evidence that dantrolene may have effects in the thermoregulation areas of the brain to thereby reduce hyperthermia.^23^ Additionally, dantrolene promptly reduces muscular rigidity, which in turn decreases hyperthermia in NMS patients and prevents myoglobinuric acute kidney injury secondary to spontaneous rhabdomyolysis common in NMS patients.^9^,^24^

Despite the inconsistencies in NMS presentation and treatment, emergency physicians need to be prepared to rapidly assess and treat patients presenting with NMS symptomatology to avoid poor outcomes. Unfortunately, previous studies lack consistency in neuroleptic dosage, scales for reporting rigidity, and a temporal sequence of NMS symptomatology, further complicating an accurate NMS diagnosis (Table 2).^10^ As our case series shows, the first patient presented with hyperthermia and rigidity typical of NMS and went on to develop serious complications, which greatly lengthened his recovery time. Complications secondary to NMS include acute kidney injury, respiratory failure, myocardial infarction, and toxic encephalopathy. Furthermore, such serious complications predict a poor prognosis. Therefore, prompt treatment should follow high suspicion for NMS and is imperative to avoid increased morbidity and mortality.

Our case series demonstrates that dantrolene may be effective in treating NMS because it affects both muscular and central nervous systems, especially if NMS was caused by neuroleptic monotherapy. Fortunately, in our case series both patients were brought in by family members who reported recent doses of neuroleptic monotherapy, which led to a faster diagnosis of NMS and treatment strategies including the use of dantrolene.

## CONCLUSION

Our case series provides emergency physicians with critical examples of NMS symptomatology that presented in an ED and the potential benefit of using dantrolene for NMS resulting from neuroleptic monotherapy. Additionally, it is our hope that our case series will contribute to the growing body of knowledge regarding NMS presentation and treatment and encourage future studies to further explore NMS diagnostic criteria and appropriate treatment regiments.

## Figures and Tables

**Table 1:** Diagnostic criteria via Diagnostic and Statistical Manual of Mental Disorders (DSM-5),^5^ Levenson,^4^ and Caroff and Mann.^6^

Source	Presentation features	Diagnostic criteria
Diagnostic and Statistical Manual of Mental Disorders (DSM-5)^5^	Exposure to dopamine antagonist within 72 hours prior to symptom development Hyperthermia (>100.4°F or >38°C on at least two occasions) Generalized rigidity Creatine kinase elevation (at least four times upper limit of normal) Changes in mental status Autonomic instability (tachycardia, diaphoresis, blood pressure elevation or fluctuation, urinary incontinence, pallor)	Presence of these cardinal features are suggestive of diagnosis
	Major: Fever Rigidity Elevated creatine phosphokinase concentration Minor: Tachycardia Abnormal arterial pressure Tachypnea Altered consciousness Diaphoresis Leukocytosis	Presence of all three major, or two major and four minor features
Caroff, Mann 1936	Major: Treatment of neuroleptics within seven days of onset (2–4 weeks for depot) Hyperthermia Muscle rigidity Exclusion of other drug-induced, systemic, or neuropsychiatric illnesses Minor: Change in mental status Tachycardia, hypertension or hypotension, tachypnea or silorrhea Tremors Incontinence Creatine phosphokinase elevation or myoglobinuria, leukocytosis, metabolic acidosis	Presence of all four major items and five minor features

**Table 2:** Previously published case reports detailing subject’s age and gender, offending agent, neuroleptic malignant syndrome presentation and treatment, and patient outcome.

Source	Subject	Offending agent	Presentation	Treatment	Outcome
Al Danaf et al. 2015^1^	60M	Risperidone	Shortness of breath, confusion, symmetrical rigidity in extremities, 37.1°C, BP 83/60 mmHg, HR 106 bpm, CPK 8450–12000	Dantrolene (initial dose 120 mg IV, 100 mg per NG tube every eight hours), Bromocriptine (5 mg per NG tube every eight hours, 10 mg per NG tube every eight hours). For fever reduction, cooling methods and low-dose lorazepam as needed.	Discharged day 17
Drews et al. 2017^11^	54M	Quetiapine and haloperidol	GCS 13 declining, diffuse lead-pipe rigidity, 102.2°F–104.7°F, CK 247 IU/L	Cooling measures, offending agents were discontinued, IV dantrolene 2.5 mg/kg tapering dose, bromocriptine 2.5 to 5 mg every eight hours.	Discharged day 29.
Saha et al. 2017^12^	19M	Olanzapine	Mutism, rigidity in extremities, high-grade fever, CPK 2467 IU/L	Fluids, offending agent was stopped, bromocriptine 5 mg/day to 15 mg/day, lorazepram 4 mg/day.	Discharged day 23 with quetiapine at low dose and gradually increased to 200 mg/day.
Ahmad et al. 2013^13^	22M	Flupentixol decanoate and Clozapine	GCS 6, increased muscular tone and joint rigidity, fever, BP 70/40, HR 168 bpm, CK 31010 units	Supportive therapy including ventilation, paralysis, intravenous fluids, antipyretics, passive cooling and sedation.	Patient developed compartment syndrome of the right forearm. Fasciotomy with debridement along with a skin harvest and was finally discharged to the psychiatric unit. Brachial plexus injury was also identified and gradually resolved in six months.
Leenhardt et al. 2017^7^	49M	Clozapine and loxapine	Confusion, muscle rigidity, 39.5–40.8°C, BP 163/90 mmHg, HR 139 bpm	Withdrawal of offending agent.	Discharged day 11.
Leenhardt et al. 2017^7^	71M	Loxapine	Recent muscle rigidity, 41.2°C/ 106.2°F, CK 562–6760 UI/L	Transferred to the intensive care unit, no NMS treatment started.	Developed multiple organ failure with secondary acute renal insufficiency requiring dialysis, metabolic acidosis, rhabdomyolysis, nosocomial pneumonia, and cardiopulmonary arrest with severe hypoxia. Died 22 days after onset.
Kuchibatla et al. 2009^14^	32M	Haloperidol depot and zuclopenthixol decanoate depot, and clozapine	Dizziness upon standing, 37.9°C to 38.5°C, BP 69/59 mmHg and 144/93 mmHg, HR 120 bpm, CK 216–521 IU/L	Offending agent stopped, vital signs normalized over four days, CK down to 152 IU/L by day 10.	Restarted on zuclopenthixol depot after an initial test dose of 100 mg intramuscularly, discharged to day hospital for monitoring, reviewed in outpatient clinic with zuclopenthixol decanoate depot, 200 mg intramuscularly weekly.
Rajamani et al. 2016^15^	43M	Risperidone	Altered sensorium, muscle rigidity, high-grade fever, CPK 1543 IU/L	Offending agent was stopped immediately, and he was treated with lorazepam, trihexyphenidyl, paracetamol, and intravenous fluids.	Discharged day 3 with monitoring of glycemic control and no antipsychotics.
Chandran et al. 2003^16^	81M	Loxapine and methotrimeprazine	Some cognitive impairment, tremor, rigidity and unsteady gait, 38.3–39.3°C, BP 124/84 mmHg, HR 128 bpm, CK 1145–2574 U/L	Offending agent was stopped, dantrolene (70 mg intravenously), after 24 hours changed to bromocriptine (2.5 mg three times daily).	Discharged five weeks, on olanzapine (2.5 mg once daily) and sertraline (25 mg once daily).
Sagud et al. 2016^17^	30F	Risperidone and haloperidol	Altered consciousness, muscular rigidity and tremor, 38.6°C, HR 123 bpm, CK 3486 U/L	Offending agents were discontinued, and she was transferred to the ICU, where she stayed for two weeks. Despite normal temp and CK, patient developed catatonia, presenting with negativism, mutism, and occasional episodes of uncontrolled motor restlessness. Electroconvulsive therapy, where she received 12 applications and her condition improved.	The patient was discharged and restarted on clozapine.

*M*, male; *BP*, blood pressure; *mmHg*, millimeters of mercury; *HR*, heart rate; *bpm*, beats per minute; *CPK*, creatine phosphokinase; *IV*, intravenous; *NG*, nasogastric; *GCS*, Glasgow Coma Scale; *IU*, international units; *L*, liter; hrs, hours; *U/ L*, units per liter; *CK*, creatine kinase; *NMS*, neuroleptic malignant syndrome; *ICU*, intensive care unit.

*M*, male; *F*, female; *CPK*, creatine phosphokinase; *CK*, creatine kinase; *HR*, heart rate; *BP*, blood pressure; *IU/L*, international units/liter; *U/L*, units per liter; *bpm*, beats per minute; *ICU*, intensive care unit.

**Table 3:** Patient one outpatient medications prior to neuroleptic malignant syndrome presentation.

Drug	Dose
Haloperidol	5 mg taken orally, daily
Clonidine	0.3 mg patch, weekly
Clonidine	0.2 mg taken orally, every 8 hours
Topiramate	50 mg taken orally, daily
Diphenhydramine	50 mg taken orally, daily
Hydroxyzine	25 mg taken orally, daily
Metoprolol succinate	100 mg taken orally, daily
Aripiprazole	5 mg taken orally, daily
Desvenlafaxine	100 mg taken orally, daily
Phenytoin	400 mg taken orally, twice daily
Amlodipine	5 mg taken orally, daily
Tamsulosin hydrochloride	0.4 mg taken orally, daily
Rosuvastatin	10 mg taken orally, daily
Cyclobenzaprine hydrochloride	10 mg taken orally, three times daily
Metoclopramide	10 mg taken orally, before meals
Eszopiclone	3 mg taken orally, before bed
Tramadol	50 mg taken orally, twice daily
Sitagliptin	100 mg taken orally, daily
Losartan-hydrochlorothiazide	100 / 25 mg taken orally, daily
Insulin aspart	25 units subcutaneous injection, before meals
Insulin detemir	50 units subcutaneous injection, twice daily

*mg*, milligrams.

**Table 4:** Patient two outpatient medications prior to neuroleptic malignant syndrome presentation.

Drug	Dose
Risperidone	2 mg taken orally, daily
Risperidone	25 mg intramuscular injection, every two weeks

*mg*, milligrams.

**Table 5:** Standard treatment pathway for neuroleptic malignant syndrome.^8^

	Treatment
Stop causative agent	Remove causative agent and any other contributing psychotropic. If NMS was due to discontinuation of dopaminergic therapy, then it should be reinstituted.
Supportive care	Maintain cardiorespiratory stability, maintain fluid balance, lower fever, lower blood pressure if elevated, prescribe heparin for prevention of venous thromboembolism, use of benzodiazepines to control agitation as necessary.
Medical therapy	
Lorazepram	1 to 2 mg IM or IV every four to six hours or diazepam 10 mg IV every eight hours.
Dantrolene	1 to 2.5 mg/kg IV are typically used in adults and can be repeated to a maximum dose of 10 mg/kg/day.
Bromocriptine	2.5 mg (through nasogastric tube) every six to eight hours are titrated up to a maximum dose of 40 mg/day
Amantadine	Initial dose is 100 mg orally or via gastric tube and is titrated upward as needed to a maximum dose of 200 mg every 12 hours.
Levodopa, apomorphine, carbamazepine, benzodiazepines	Have been used with some anecdotal success
Electroconvulsive therapy	Efficacy in treating malignant catatonia and reports of parkinsonism improving with ECT. ECT is generally reserved for patients not responding to other treatments or in whom nonpharmacologic psychotropic treatment is needed.

*NMS*, neuroleptic malignant syndrome; *mg*, milligram; *IM*, intramuscularly; *IV*, intravenously; *kg*, kilogram(s); *ECT*, electroconvulsive therapy.
